# A Perfusion Index-Based Evaluation and Comparison of Peripheral Perfusion in Sevoflurane and Isoflurane Anaesthesia: A Prospective Randomised Controlled Trial

**DOI:** 10.5152/TJAR.2023.21435

**Published:** 2023-04-01

**Authors:** Neeraja Ajayan, Jayakumar Christudas, Linette Morris, Oommen Mathew, Ajay Prasad Hrishi

**Affiliations:** 1 Neurocritical Care, Addenbrookes Hospitals, Cambridge University Hospitals, Cambridge, United Kingdom; 2 Department of Anaesthesiology, Trivandrum Medical College, Trivandrum, India; 3 University of Kerala, Trivandrum, India; 4 Division of Neuroanaesthesia, Department of Anaesthesiology, Sree Chitra Tirunal Institute for Medical Sciences and Technology, Trivandrum, India

**Keywords:** Isoflurane, perfusion index, peripheral perfusion, sevoflurane, vasomotor tone

## Abstract

**Objective:**

Perfusion index has shown to be helpful in the operative and critical care settings to monitor peripheral tissue perfusion. Randomised controlled trials quantifying different agents’ vasodilatory properties using perfusion index has been limited. Therefore, we undertook this study to compare the vasodilatory effects of isoflurane and sevoflurane using perfusion index.

**Methods:**

This is a pre-specified sub-analysis of a prospective randomised controlled trial on the effects of inhalational agents at equipotent concentration. We randomly allocated patients scheduled for lumbar spine surgery to either isoflurane or sevoflurane groups. We recorded values of perfusion index at age-corrected 1 Minimum Alveolar Concentration (MAC) concentration at baseline, pre- and post-application of a noxious stimulus. The primary outcome of interest was the measure of vasomotor tone with perfusion index, and the secondary outcomes which were analysed were mean arterial pressure and heart rate.

**Results:**

At age-corrected 1.0 MAC, there was no significant difference in the pre-stimulus haemodynamic variables and perfusion index between both groups. During the post-stimulus period, there was a significant increase in heart rate in the isoflurane group compared to the sevoflurane group, with no significant difference in the mean arterial pressure values between both groups. Though the perfusion index decreased during the post-stimulus period in both groups, there was no statistically significant difference between the 2 groups (*P* = .526, repeated-measures analysis of variance).

**Conclusion:**

In a steady state of age-corrected 1.0 MAC, isoflurane and sevoflurane had a similar perfusion index before and after a standardised nociceptive stimulus, which suggests that both of these agents have similar effect on peripheral perfusion and vasomotor tone.

**Main Points**

- Changes in the perfusion index during anaesthesia have been described; however, randomised controlled trials quantifying different agents’ vasodilatory properties using perfusion index has been limited.
- We compared peripheral perfusion and vasomotor tone at equipotent doses of isoflurane and sevoflurane using perfusion index.
- In a steady state of age-corrected 1.0 MAC, isoflurane and sevoflurane had a similar perfusion index before and after a standardised nociceptive stimulus. These findings suggest that both of these agents have a similar effect on peripheral perfusion and vasomotor tone.

## Introduction

Perioperative tissue hypoperfusion and resultant hypoxia are major contributing factors of poor outcomes in surgical population. Surgical stress response could result in increased oxygen demand and failure to meet this can lead to consequent tissue hypoxia.^1-3^ With increasing severity and duration of tissue hypoxia, detrimental complications such as organ injury or death could result.^1^

During the intra-operative period, the pharmacological effects of the anaesthetic agents could affect the homeostasis, which has a significant impact on the outcomes of the patients.^4^ Advanced and continuous monitoring of systemic haemodynamics is invasive and requires dedicated devices and expertise. However, standard perioperative cardiovascular monitoring may fail to detect tissue hypoxia.^5,6^ This results in ‘under monitoring’ of patients with an unknown cardiovascular risk, resulting in their increased predisposition to haemodynamic compromise.^7^ Multiple studies and meta-analyses have shown poor outcomes in surgical patients who had even a short duration of haemodynamic instability.^8^ This necessitates 2 things: first, comprehending the effects of different anaesthetic agents on homeostasis to optimise its administration ,and second, the need for a feasible, continuous, non-invasive and cost-effective way to monitor tissue perfusion.

Perfusion index (PI) has shown to be helpful in the operative and critical care settings to monitor peripheral tissue perfusion.^9^ Changes in the PI during anaesthesia have been described; however, randomised controlled trials quantifying different agents’ vasodilatory properties using the same technique has been limited. Isoflurane and sevoflurane are 2 commonly used volatile anaesthetics agents in spine surgeries. Therefore, we undertook this study to compare the vasodilatory effects of isoflurane and sevoflurane using PI. A quantitative comparison of their effects on the vasomotor tone and peripheral perfusion can help us choose the ideal inhalational anaesthetics in our clinical practice.

## Methods

This is a pre-specified sub-analysis of a prospective randomised comparative study on the effects of inhalational agents at equipotent concentration. After obtaining approval from the Institutional Ethics committee, consenting patients between 18 and 60 years scheduled for elective lumbar disc surgery were included in the study. Patients with American Society of Anaesthesiologists (ASA) physical status III and higher, those receiving medications that can affect vasomotor tone such as vasopressors and anti-hypertensive drugs, medications acting on the autonomic nervous system including beta-blockers and vagolytics, presence of systemic or peripheral vascular disease, diabetes mellitus, neurological or psychiatric ailments and history of substance abuse were excluded from the study. Motion artefacts in the plethysmograph wave, entropy values >70 and haemodynamic derangements (hypotension defined as a systolic blood pressure <90 mmHg or mean arterial pressure <65 mm Hg, hypertension defined as >20% of baseline blood pressure, bradycardia defined as heart rate <50 per minute, and tachycardia defined as heart rate >140 per minute) were the reason for withdrawal of the subject from the study.

Using a web-based response, a random-permuted block randomisation algorithm randomly allocated 20 patients to each group. Allocation concealment was ensured with opaque serially numbered envelopes containing protocol with the name of the agent to be used. Premedication drugs such as anxiolytics and anticholinergics were avoided in the study population.

In the operating room, standard pre-induction monitoring, comprising electrocardiography (ECG), non-invasive blood pressure (NIBP) and pulse oximetry (SpO_2_), were attached. The entropy electrode was applied to the patient's forehead and connected to the monitor. General anaesthesia was induced with IV propofol 2 mg.kg^-1^, and tracheal intubation was facilitated with IV succinylcholine 2 mg.kg^-1^. In addition, lignocaine 2 mg.kg^-1^ was administered to blunt the autonomic responses to intubation. The peripheral nerve stimulator electrodes were placed over the ulnar nerve on the volar aspect of the distal forearm. A train-of-four (TOF) count of 0 was ensured before intubation using a neuromuscular monitor device. After intubation, mechanical ventilation with air: O_2_ 50%: 50% was initiated. End-tidal CO_2_ monitoring was instituted to ensure normocarbia. Temperature probe was placed in the nasopharynx, and normothermia was ensured throughout the study period. Perfusion index was measured in the index finger using pulse oximetry (Beneview T8, Mindray, China). The patient was kept supine until the end of the study period, and the hands were placed in a neutral position. We placed the pulse oximeter in the arm opposite to where the non-invasive blood pressure cuff was placed. The room's ambient temperature was constant at approximately 23 to 25°C throughout the study period. An upper body warming blanket was placed after prone positioning, and forced-air warming (Bair Hugger, model no. 505; Arizant Healthcare Inc., Eden Prairie, MN, USA) was used to maintain the patient’s body at a set temperature of 38 C. I.V. fluid administration was standardised to 5 mL.kg^-1^.hr^-1^ of normal saline solution in both groups till the end of the study period.

At this juncture, the volatile anaesthetics agent was introduced by over-pressurisation to target an age-corrected MAC of 1.0. The gas analyser continuously monitored end-tidal anaesthetics concentration (GE Datex Ohmeda S5 Anesthesia Monitor). The noxious stimulus was provided after 20 minutes to ensure the steady-state concentration of the volatile agent and to avoid the residual effects of propofol. Next, the noxious stimulus was provided to the subject by tetanic stimulation (square-wave, 70 mA stimulus, 30-sec duration at 50 Hz), and the post-noxious stimulus study parameters were obtained. Opioids were administered after the recording of the post-stimulus values.

The study parameters, namely heart rate (HR), mean arterial pressure (MAP), and PI, were recorded at 3 pre-defined time points:

1. Baseline: before anaesthetics induction
2. Pre-noxious stimulus: recorded after induction of anaesthesia, before providing noxious stimulus
3. Post-noxious stimulus: recorded after application of noxious stimuli

During the study period, if the entropy values were greater than 70, additional sedatives or analgesics would be administered. If any of the patients encountered haemodynamic derangements during the study period, it would be promptly managed.

### Statistical Analysis

The primary outcome of interest was the measure of vasomotor tone with PI, and the secondary outcomes which were analysed were MAP and HR. There are no prior studies comparing the perfusion index between isoflurane and sevoflurane. We initially did a pilot study containing 20 patients (10 per group), wherein the pre-stimulation PI values (mean [SD]) were 4.9 (0.8) for the sevoflurane group and 4.1 (1) for the isoflurane group. We estimated the sample size of 20 patients per group for this study using a 2-sided *t*-test, with a power of 80%, and a significance level of 5%. Statistical analyses were performed using the SPSS 17.0 version (SPSS Inc., Chicago, IL, USA). Data are presented as frequency for categorical variables and mean ± SD or median (interquartile range) for continuous variables. The Shapiro–Wilk test was used to test the normalcy of distribution for the variables. The comparison of categorical variables between the 2 groups was made using the Fisher exact test. The Student's *t*-test or the Mann–Whitney *U*-test (depending on the distribution) was used for continuous variables. Repeated-measures analysis of variance (RM-ANOVA) was used to compare consecutive measurements of the PI, MAP, and HR values at different time points between the 2 groups. A *P*-value < .05 was considered to be statistically significant.

## Results

**Figure 1.**
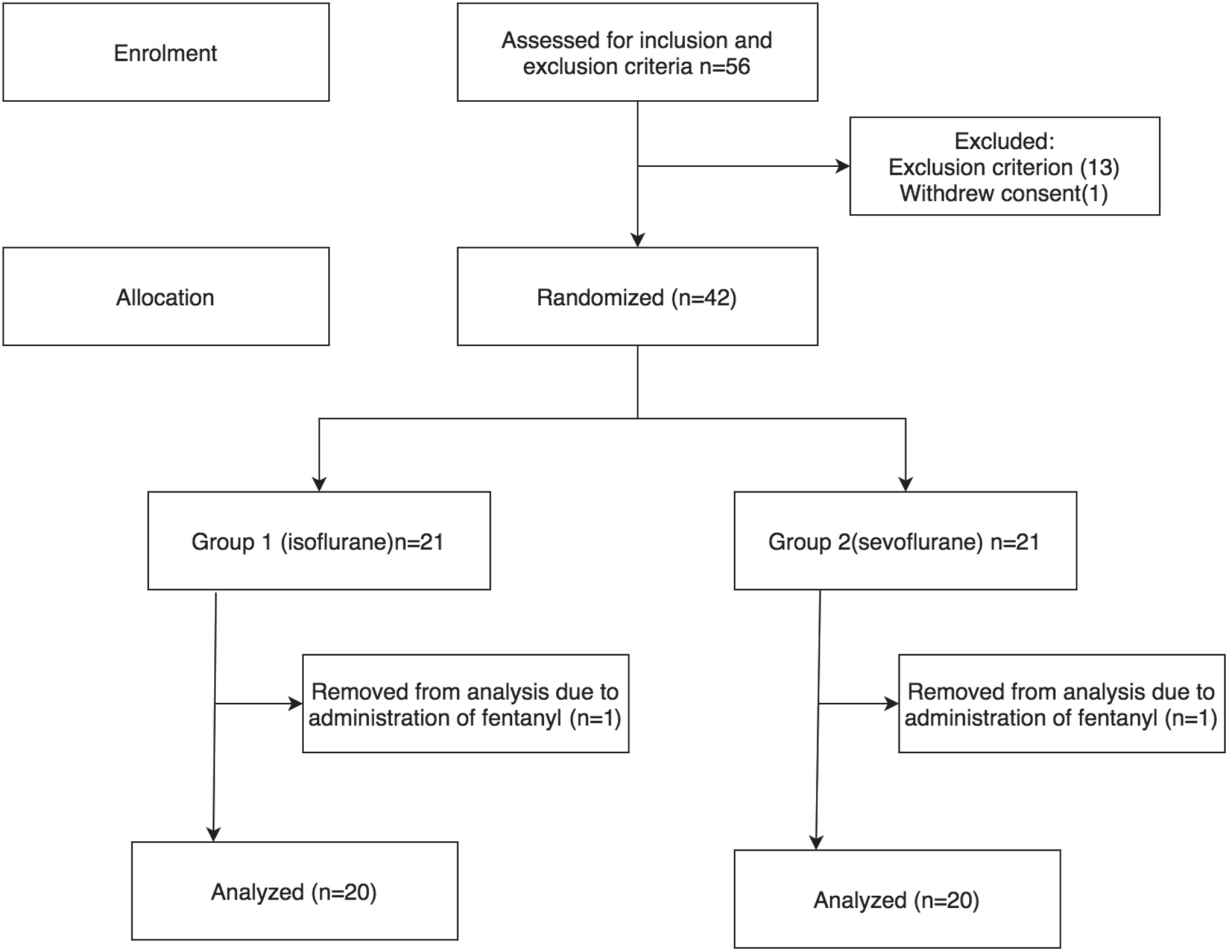
CONSORT flow diagram for the recruitment and allocation of subjects in the study.

A total of 56 patients presenting for spinal surgery were recruited for the study. A total of 12 patients were ineligible based on the exclusion criterion (Figure 1). One patient withdrew consent and was excluded from the study. Therefore, 21 subjects were included in each group. One patient from each group was excluded from the study because of the administration of fentanyl. Thus, data of 20 subjects in each group were taken for final analysis (Figure 1). The demographic characteristics and perfusion indices were comparable between the groups (Table 1).

**Figure 2.**
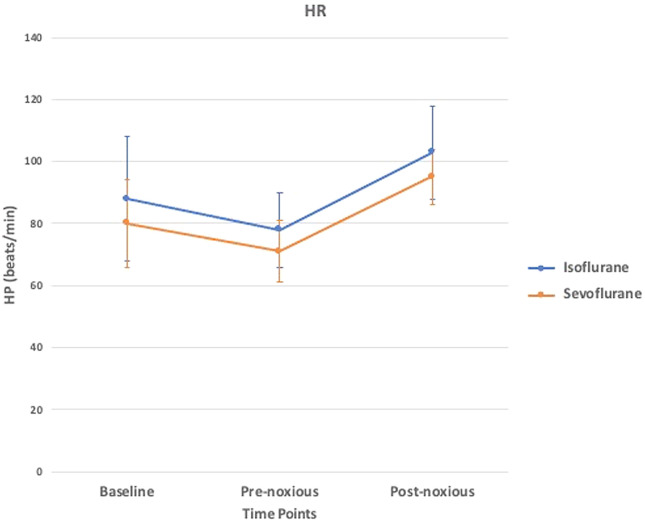
Graph showing trends of HR, MAP and PI at different time points. Values are expressed as mean and 95% confidence interval for HR and MAP. Values are expressed as median and 95% confidence interval for PI.

**Figure 3.**
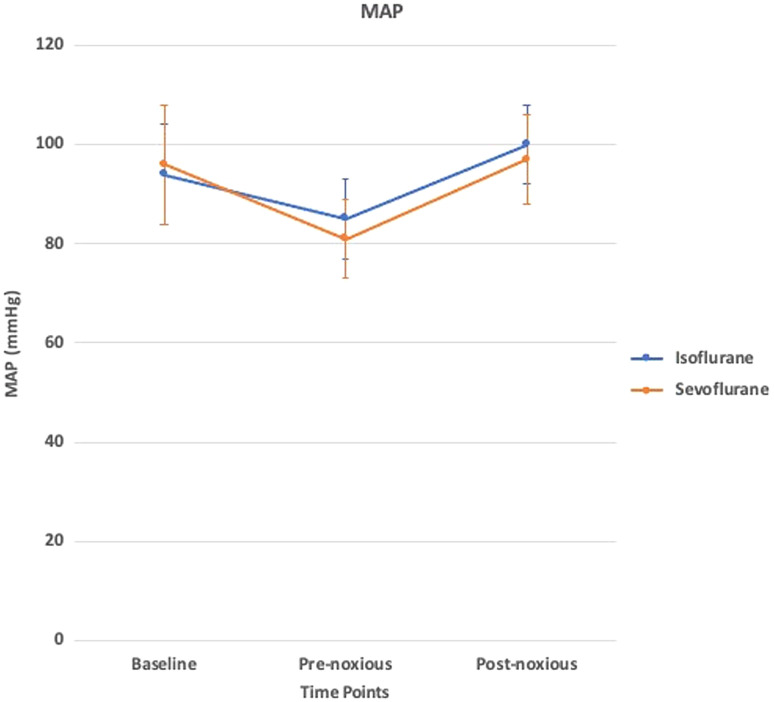
Graph showing mean arterial pressure (MAP) trends at different time points. Values are expressed as mean and 95% CI for MAP. *P* value = .056 by repeated-measures analysis of variance.

**Figure 4.**
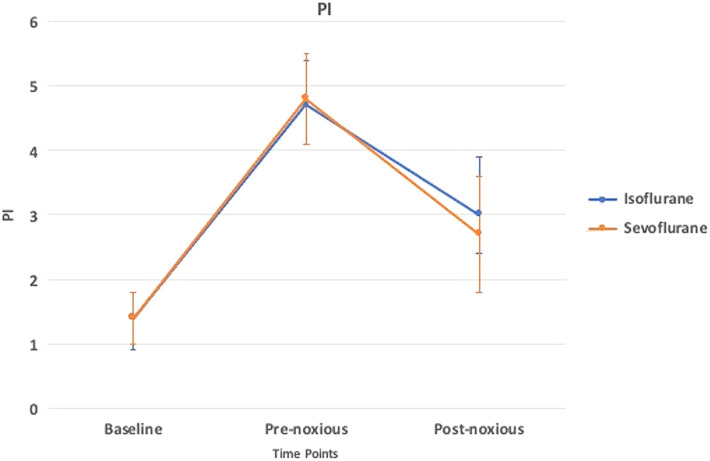
Graph showing perfusion index (PI) trends at different time points. Values are expressed as median and 95% CI for PI. *P* value = .526 by repeated-measures analysis of variance.

The baseline, pre-noxious and post-noxious variables were evaluated for both groups (Table 2, Figures 2-4) The baseline haemodynamic values before the induction were comparable between the 2 groups (Table 2, Figure 3, 4).

### Primary Outcome: Perfusion Index

There was no statistically significant difference in the pre-stimulus perfusion index between the isoflurane and sevoflurane groups [4.71 (4-5.8), 4.80 (4.2-5.5); *P* = 0.891]. Though the perfusion index decreased during the post-stimulus period in both groups, there was no statistically significant difference between the 2 groups (*P* = .167) (Table 2). The median perfusion index was 2.70 (2.1-3.6) in the sevoflurane group compared to 3.00 (2.3-4.2) in the isoflurane group (*P* = .63). With RM-ANOVA, there was no statistically significant difference between the 2 groups with regard to both group and time effects (*P* = .526) (Figure 2).

### Secondary Outcomes: Heart Rate and Mean Arterial Pressure

At age-corrected 1.0 MAC, there was no significant difference in the pre-stimulus haemodynamic variables between both the groups (HR *P* = .061, MAP *P* = .172). During the post-stimulus period, there was a significant increase in HR in the isoflurane group compared to the sevoflurane group (103.11 ± 15.17 vs. 95.42 ± 9.08; *P* = .047) (Table 2). However, there was no significant difference in the MAP values between both groups during the post-stimulus period (*P* = .390) (Table 2). The MAP and HR values of participants in both the groups were similar (MAP *P* = .056 and HR *P* = .422 by RM-ANOVA) when group and time effects were examined (Figures 3 and 4).

## Discussion

The aim of our study was to compare the effects of age-corrected 1.0 MAC of isoflurane and sevoflurane on vasomotor tone and peripheral perfusion. We have found that isoflurane and sevoflurane had comparable effects on vasomotor tone and peripheral perfusion both before and after noxious stimulus.

In conventional clinical practice, global haemodynamic measurements such as blood pressure, HR, markers of fluid responsiveness such as pulse pressure variation and systolic pressure variation, and indicators of organ perfusion such as urine output and serum lactate are considered surrogates of tissue perfusion.^1,5^ The major caveat is that even though these variables provide a certain level of information on tissue perfusion, it is not precise. Also, in the case of hypotension, vital organ perfusion is maintained at the expense of decreased perfusion of other tissue beds like the gut and skin. The standard monitoring can’t monitor these organs at stake.^1,6^ Thus, monitoring the perfusion of these tissues like skin could have potential benefits. First, it would help in the early detection of decreasing tissue perfusion. Second, non-invasive continuous monitoring of peripheral perfusion is easier and feasible and requires lesser expertise.^6^

One way to measure peripheral perfusion is with PI, derived from the photoelectric plethysmographic waveform. Using pulse oximetry, PI is calculated as the ratio between the pulsatile (which reflects the arterial component) and non-pulsatile (which reflects the other tissues) signals of absorbed light and is calculated independently of the patient's oxygen saturation.^9^ Any alteration in the peripheral perfusion is accompanied by a concurrent change in the pulsatile component. Since the non-pulsatile component does not change, the ratio changes.^5^ Sympathetic tone is the primary determinant of PI. Peripheral vasodilation causes an increase in PI, whereas vasoconstriction causes a decrease in the values; thus, PI is a direct indicator of peripheral perfusion. It can be used as a surrogate for quantitative measurement of vasomotor tone produced by drugs, including anaesthetics agents.^10,11^ Multiple studies have demonstrated the utility of using this to determine successful sympathectomy in regional anaesthesia.^12,13^

Volatile anaesthetic agents influence peripheral perfusion predominantly via 2 mechanisms. The temperature at which a particular response is generated is called as threshold temperature. The threshold for vasoconstriction which is normally 36.5°C, is reduced by around 2°-4°C during anaesthesia in a dose-dependent way.^14^ As a response to this, the acral arteriovenous shunt dilatation increases around tenfold causing increased peripheral perfusion.^15^ Second, volatile anaesthetic agents increase peripheral perfusion by causing peripheral vasodilation.^3,16,17^ Ryu et al.^10^ in their study comparing PI between desflurane and sevoflurane, found an increase in PI at 1 MAC of both the agents. Treschan et al^16^, in their study, evaluated tissue oxygenation by tonometry in general and epidural anaesthesia and found that both produced an increase in tissue oxygenation compared to baseline values.

Our study used PI to evaluate the differences in peripheral perfusion produced by isoflurane and sevoflurane. Systemic vascular resistance (SVR) can accurately predict vasodilatory properties. However, it is invasive and expensive and requires high levels of expertise.^7,18^ We demonstrated that the effects of both isoflurane and sevoflurane are similar on the PI and hence on vasomotor tone. In our study, the median baseline PI was identical in both groups. After induction, the PI increased in both the groups; however, there was no significant difference between them. The increase in PI post-induction is a direct indicator of both agents' vasodilatory effect. Post-stimulation, though the values of PI reduced compared to that of pre-stimulus, the difference remained statistically non-significant between the groups.

All volatile anaesthetics induce hypotension with dose and agent-dependent effects on the central and autonomic nervous system by reducing the SVR and depression of myocardial contractility.^4^ Malan et al^19^ found that the cardiovascular effects of sevoflurane and isoflurane were similar both quantitatively and qualitatively. However, in their study, systemic vascular resistance (SVR) was maintained at 1 MAC and decreased only at higher concentrations of 1.5 and 2 MAC. Rodig et al^18^ in their study stated to exclude effects on myocardial contractility, administered sevoflurane and isoflurane during cardiopulmonary bypass and found that both the agents had a similar impact on systemic vascular resistance index (SVRI). They also found that SVRI did not change significantly from baseline at 1 MAC. Moreover, a reduction in SVRI was noted only at 3% sevoflurane and 1.8% isoflurane. However, in our study, we found an increase in PI at 1 MAC. Though it cannot be conclusively deduced from our study, this increase in peripheral perfusion at 1 MAC of isoflurane could be attributed to direct vasodilatory properties of isoflurane and acral arteriovenous dilatation as a part of reduction of thermoregulatory vasoconstriction threshold. This is further purported by a study which found that isoflurane produced a non-linear dose-dependent reduction in vasoconstriction threshold, and at 1 MAC, the threshold was reduced by approximately 4°C.^17^

Our findings regarding the haemodynamic parameters such as HR and MAP were similar to other studies, showing that sevoflurane's cardiovascular effects were similar to those of isoflurane.^19^ Malan et al^19^ showed that these agents were comparable in their cardiac effects. However, in their study, HR increased only at 1.5 and 2.0 MAC and not at 1.0 MAC. However, this wouldn’t have much significance in the clinical setting. Our findings are similar to Chen et al.^20^ who found that though sevoflurane and isoflurane had similar alterations in blood pressure during maintenance, and there was an increased incidence of tachycardia after surgical incision in patients receiving isoflurane. Frink et al^21^ also found that though both the agents produced similar blood pressure, the HR response to skin incision was greater in patients administered isoflurane. Isoflurane is thought to depress parasympathetic activity more than sympathetic activity and hence, the baroreflex response of tachycardia consequent to hypotension would be predominant.^22-25^ Nishiyama^22^ found that increasing concentration of isoflurane caused hypotension, and tachycardia which was concurrent with increased plasma concentrations of epinephrine and norepinephrine. However, an increase in sevoflurane concentration induced only hypotension and a decrease in plasma epinephrine concentration.

We studied haemodynamic parameters and perfusion index to assess whether agents with similar effects on MAP and SVR would produce comparable effects on the perfusion index. In our study, both sevoflurane and isoflurane produced similar effects on MAP and PI which elucidates that agents with similar effects on SVR would produce similar perfusion index. Ryu et al^10^ compared the PI of sevoflurane and desflurane, 2 agents with different effects on SVR. They concluded that desflurane produced a higher PI and substantially lower MAP, which implies more potent vasodilatory properties of desflurane. This study suggests that agents with different effects on MAP would have varied effects on PI. In contrast, Kowalczyk et al^26^ compared desflurane and propofol and found that though there was a significant difference in MAP between the agents, the PI in both groups was comparable. This would indicate that agents with same effects on MAP and SVR could produce dissimilar PI. While there was a strong correlation between PI and the end-expiratory concentration of desflurane, there was no correlation between PI and the predicted plasma propofol concentration. This finding is interesting because of 2 reasons. First, it shows that changes in PI tracked changes in MAP and CO during general anaesthesia.^7^ Second, there is a dose-dependent reduction in MAP and SVR with propofol.^27^ If PI was exclusively dependent on MAP and SVR, there should have been a correlation between PI and propofol concentration. These studies show that PI, though dependent on SVR and MAP, is not exclusively determined by it. The contrasting reports of the aforementioned studies^10,26^ including our study warrant research comparing PI of agents with similar and dissimilar effects on haemodynamics to assess whether PI is beyond the influence of systemic haemodynamics.

There is only limited literature comparing the peripheral perfusion effects of general anaesthetic agents.^10,26,28^ Agerskov et al^29^ concluded that low intraoperative peripheral PI was associated with severe postoperative complications or death in acute high-risk surgical patients and suggested that PI should be investigated to guide intraoperative haemodynamic management. Genderen et al^30^ demonstrated that abnormal peripheral perfusion parameters were associated with severe complications following abdominal surgery. Interestingly, in both the above studies, the results were independent of systemic haemodynamics. Perioperative haemodynamic optimisation is usually based on macrocirculatory parameters such as MAP; however, these studies suggest that monitoring of PI is a practical and valuable haemodynamic monitoring modality to reduce post-operative complications. It has also been recommended that the addition of perfusion parameters to goal-directed therapy algorithms could help guide the clinician to the correct intervention rather than relying only on macrocirculatory parameters.^31^ Differentiating the effects of anaesthetic agents on tissue perfusion and studying their impact on outcomes is essential as microvascular alterations may have a crucial role in post-operative complications. Further studies comparing the effect of anaesthetic agents on peripheral perfusion and microcirculation would throw light on this.

### Limitations

Our study results have to be cautiously interpreted in the background of PI as a surrogate quantitative measurement of vasomotor tone and peripheral perfusion. Lima et al^32^ found high inter-individual variability of PI with values ranging from 0.3 to 10%, with a median of 1.4%, thus contributing to a skewed distribution in the normal population. Because of this, PI is more useful in trend monitoring for follow-up rather than being used as a single cross-sectional measure.^33^

We compared 2 agents commonly used in the perioperative setting in the Southeast Asian subcontinent. Moreover, studies have compared PI for desflurane, sevoflurane, and propofol, the widely used anaesthetic agents. However, isoflurane is not commonly used in an intraoperative setting in developed countries and is slowly being phased out by agents such as sevoflurane and desflurane. Nevertheless, the advanced anaesthetic conserving device (AnaConDa) uses isoflurane and sevoflurane to sedate patients in critical care units. We believe that the study on PI of these 2 agents is beneficial in the background of this revamped interest in a critical care setting to adopt volatile anaesthetics as sedatives. Our sample size was calculated based on a pilot study with 10 patients. Therefore, further studies with a larger sample size are required to corroborate that isoflurane and sevoflurane have the same effects on peripheral perfusion.

We monitored temperature with a nasopharyngeal probe to ensure normothermia and took additional precaution of using forced air warming to a set temperature of 38 degree C. The study period lasted 30 minutes to 1 hour after the induction and during this initial period, the body heat redistributes from the central compartment to the periphery *via* vasodilation.^14^An additional skin temperature monitoring in the peripheries would have been beneficial to preclude effects of this redistribution of heat on perfusion index in the study.

## Conclusion

In a steady state of age-corrected 1.0 MAC, isoflurane and sevoflurane had a similar perfusion index before and after a standardised nociceptive stimulus. These findings suggest that both these agents have a similar effect on peripheral perfusion and vasomotor tone. However, further studies to elucidate whether these vasodilatory properties of inhalational anaesthetic agents are beneficial for tissue oxygenation and microcirculation are required.

## Figures and Tables

**Table 1.** Demographic Details of Patients in Both Groups

Parameters	Group 1 (Isoflurane)	Group 2 (Sevoflurane)	*P*
Age (years)	45.32 ± 12	41.20 ± 14	.338
Male/female (n)	12/8	10/10	.751
Height (cm) [mean ± SD]	159.17 ± 12	155.45 ± 10	.259
Weight (kg) (mean ± SD)	69.04 ± 15	73.22 ± 18	.449
ASA PS (I/II)	18/2	17/3	.632
Hypertension (n)	2	2	1
Bronchial asthma (n)	0	1	1

ASA PS, American Society of Anesthesiologists Physical status grade

**Table 2.** Haemodynamic Parameters and Perfusion Indices of Both the Groups

Variables	Group 1 (Isoflurane)	Group 2 (Sevoflurane)	*P*
*Baseline*			
HR (mean ± SD)	88.57 ± 20.01	80.41 ±14.11	.151
MAP (mean ± SD)	94.10 ± 10.56	96.22 ±12.36	.570
PI [median (IQR)]	1.40 (1-1.7)	1.41 (0.9-1.8)	.810
*Pre-noxious stimulus*			
HR (mean ± SD)	78.23 ±12.21	71.45 ±10.13	.061
MAP (mean ± SD)	85.37 ±8.05	81.88 ±8.10	.172
PI [median (IQR)]	4.71 (4-5.8)	4.80 (4.2-5.5)	.890
*Post-noxious stimulus*			
HR (mean ± SD)	103.11 ±15.17	95.42 ±9.08	.047^*^
MAP (mean ± SD)	100.36 ±8.14	97.12 ±9.86	.390
PI [median (IQR)]	3.00 (2.3-4.2)	2.70 (2.1-3.6)	.167

^*^ *P* < .05 is considered statistically significant.

HR, heart rate; IQR, interquartile range; MAP, mean arterial pressure; PI, perfusion index.
